# Pharmacokinetic Characterization of the DDAH1 Inhibitors ZST316 and ZST152 in Mice Using a HPLC-MS/MS Method

**DOI:** 10.3390/molecules27031017

**Published:** 2022-02-02

**Authors:** Arduino A. Mangoni, Tommaso Ceruti, Roberta Frapolli, Massimo Russo, Stefania Fichera, Massimo Zucchetti, Sara Tommasi

**Affiliations:** 1 Discipline of Clinical Pharmacology, College of Medicine and Public Health, Flinders University, Bedford Park, SA 5042, Australia; 2 Department of Clinical Pharmacology, Flinders Medical Centre, Southern Adelaide Local Health Network, Bedford Park, SA 5042, Australia; sara.tommasi@sa.gov.au; 3 Laboratory of Cancer Pharmacology, Department of Oncology, Istituto di Ricerche Farmacologiche Mario Negri IRCCS, 20156 Milan, Italy; tommaso.ceruti@marionegri.it (T.C.); roberta.frapolli@marionegri.it (R.F.); stefania.fichera@guest.marionegri.it (S.F.); massimo.zucchetti@marionegri.it (M.Z.); 4 Laboratory of Cancer Metastasis Therapeutics, Department of Oncology, Istituto di Ricerche Farmacologche Mario Negri IRCCS, 20156 Milan, Italy; massimo.russo@marionegri.it

**Keywords:** dimethylarginine dimethylaminohydrolase-1 inhibitors, ZST316, ZST152, pharmacokinetics, safety, mice

## Abstract

The pharmacokinetic profile of ZST316 and ZST152, arginine analogues with inhibitory activity towards human dimethylarginine dimethylaminohydrolase-1 (DDAH1), was investigated in mice using a newly developed HPLC-MS/MS method. The method proved to be reproducible, precise, and accurate for the measurement of the compounds in plasma and urine. Four-week-old female FVB mice received a single dose of ZST316 and ZST152 by intravenous bolus (30 mg/Kg) and oral gavage (60 mg/Kg). ZST316 Cmax was 67.4 µg/mL (intravenous) and 1.02 µg/mL (oral), with a half-life of 6 h and bioavailability of 4.7%. ZST152 Cmax was 24.9 µg/mL (intravenous) and 1.65 µg/mL (oral), with a half-life of 1.2 h and bioavailability of 33.3%. Urinary excretion of ZST152 and ZST316 was 12.5%–22.2% and 2.3%–7.5%, respectively. At least eight urinary metabolites were identified. After chronic intraperitoneal treatment with the more potent DDAH1 inhibitor, ZST316 (30 mg/Kg/day for three weeks), the bioavailability was 59% and no accumulation was observed. Treatment was well tolerated with no changes in body weight vs. untreated animals and no clinical signs of toxicity or distress. The results of this study show that ZST316 has a favorable pharmacokinetic profile, following intraperitoneal administration, to investigate the effects of DDAH1 inhibition in mice.

## 1. Introduction

The isoform 1 of the enzyme dimethylarginine dimethylaminohydrolase (DDAH1) plays a critical role in the metabolism of the endogenous methylated arginines, asymmetric N^G^,N^G^-dimethyl-L-arginine (ADMA) and N^G^-monomethyl-L-arginine (NMMA), into L-citrulline and dimethylamine [1,2,3]. As both ADMA and NMMA are potent endogenous inhibitors of the three isoforms of nitric oxide (NO) synthase (NOS), DDAH1 regulation significantly influences NO synthesis [4,5]. In particular, the downregulation of DDAH1, and the consequent accumulation of the substrates ADMA and NMMA, leads to NOS inhibition [3,4,6,7,8]. The pharmacological inhibition of DDAH1 is emerging as an attractive therapeutic strategy in disease states, e.g., septic shock and cancer, that are characterized by excessive local and/or systemic NO synthesis, primarily mediated by the inducible NOS isoform (iNOS) [9,10,11,12,13,14].

**Figure 1:**
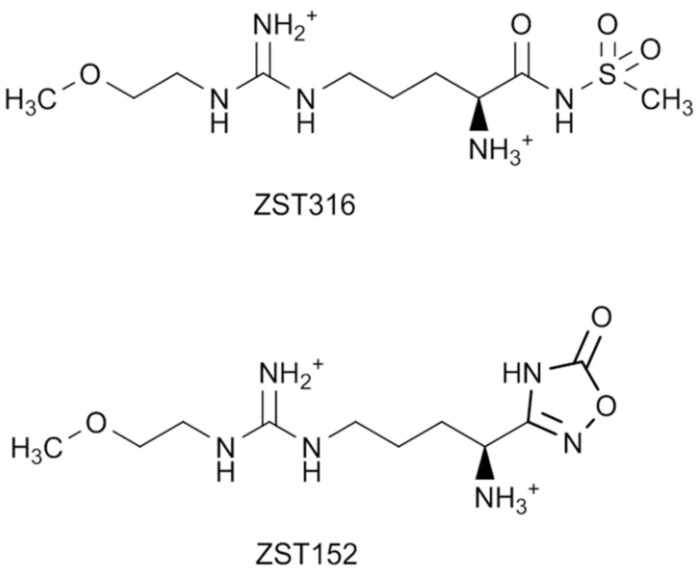
Chemical structures of the DDAH1 inhibitors ZST316 and ZST152.

We have previously synthesized a new series of arginine analogues incorporating carboxylate bio-isosteric functions, with significant inhibitory activity towards human recombinant DDAH1, stably expressed in HEK293T cells. Compound 10a (ZST316), characterized by an acylsulfonamide isosteric replacement of the carboxylate, showed a 13-fold greater inhibitory activity (IC_50_: 3 μM; *K*_i_: 1 μM) towards DDAH1 when compared to the reference DDAH1 inhibitor, L-257. Another compound of the series, the oxadiazolone 14b (ZST152), was also effective (IC_50_: 18 μM; *K*_i_: 7 μM) (Figure 1) [15]. ZST316 and ZST152 have been shown in vitro to suppress vasculogenic mimicry, an alternative neo-vascularization pathway driven by iNOS and DDAH1 that increases metastatic potential and adverse outcomes, in triple-negative breast cancer cells [11,16]. In these experiments, ZST316 and ZST152 did not significantly degrade in the culture media and were able to successfully penetrate the cell membrane of cancer cells and inhibit DDAH1 intracellularly [17]. 

Given the promising results obtained in vitro, and with a view to conduct studies characterizing the pharmacological profile in vivo, we developed a new HPLC-MS/MS method to measure ZST316 and ZST152 concentrations in the plasma and urine of mice. The method was successfully used in the comprehensive pharmacokinetic characterization of the two compounds, determining their safety profile and the most appropriate route of administration and dosing regimen for in vivo efficacy studies. A similar approach has been successfully used by other groups [18,19,20,21,22,23].

## 2. Results

### 2.1. HPLC-MS/MS

**Figure 2:**
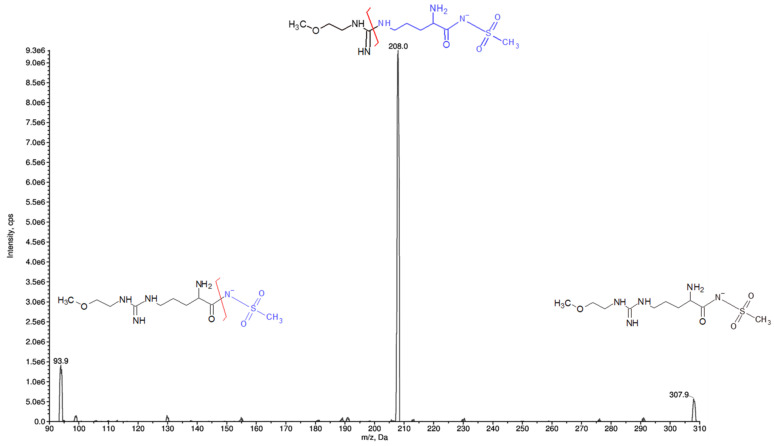
SRM fragmentation pattern of ZST316 obtained by tandem mass spectrometry.

**Figure 3:**
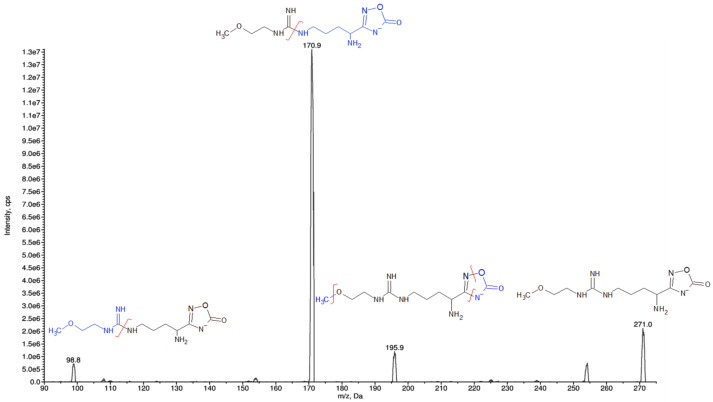
SRM fragmentation pattern of ZST152 obtained by tandem mass spectrometry.

**Figure 4:**
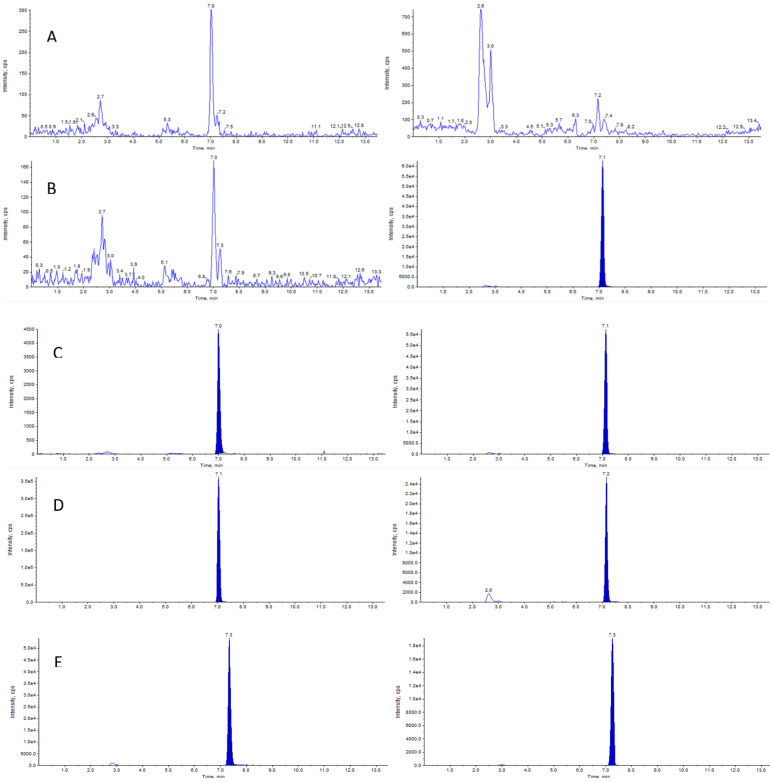
Representative chromatograms of a double blank plasma sample (**panel** (**A**)), blank with internal standard IS (ZST152, **panel** (**B**)), LLOQ of ZST316 with IS (ZST152, **panel** (**C**)), and of unknown study plasma samples analyzed for ZST316 with IS (ZST152, **panel** (**D**)) and ZST152 with IS (ZST316, **panel** (**E**)).

Analyte detection and quantification were carried out by tandem mass spectrometry (MS/MS) using the following mass transitions: 308.0 > 208.0 m/z for ZST316, and 271.0 > 170.9 m/z for ZST152. The detailed selected reaction monitoring (SRM) reporting the full fragmentation pattern of ZST316 and ZST152 obtained by MS/MS is shown in Figure 2 and Figure 3. Examples of chromatograms including a blank plasma and the lower limit of quantitation (LLOQ) samples are shown in Figure 4.

### 2.2. Development of the Analytical Method

#### 2.2.1. Plasma

The extraction recovery, established on QCs at the concentrations of LLOQ (i.e., 5 ng/mL), at 15 and 250 ng/mL, fell in the range 88.9%–94.6% and 87.9%–117.5% for ZST316 and ZST152, respectively. The LLOQ in plasma was 5.0 ng/mL for both compounds, with precision of 7.1% and 4.1% and accuracy of 95.8% and 106.1% (*n* = 6) for ZST316 and ZST152, respectively (Table 1). We decided to validate this value as LLOQ even though the limit of detection (LOD) reached in the initial set up phase of the methods was established for both compounds at 1 ng/mL (S/N > 4.0). However, such sensitivity was not required as the expected range of activity (IC_50_) was markedly higher than the LLOQ value for both compounds. The linearity of the method, evaluated over three different analytical runs in the concentration range 5.0–500.0 ng/mL, was acceptable for both analytes, with an average coefficient of determination (R^2^) of 0.990 and 0.992 for ZST316 and ZST152, respectively. The method proved to be reproducible, precise, and accurate as demonstrated by the intra-day and inter-day results from QC plasma samples of ZST316 and ZST152 (Table 2 and Table 3). Both ZST316 and ZST152 were stable in frozen plasma for at least one month at −20 °C. No chromatographic carry-over effect was observed injecting a blank sample after the highest standard calibration point. The method was considered suitable for preclinical pharmacokinetic investigations.

#### 2.2.2. Urine

The linearity of the method, across three separate analytical runs in the concentration range 1.0–50.0 µg/mL for both analytes, was demonstrated by the mean determination coefficient (R^2^), 0.990 and 0.997 for ZST316 and ZST152, respectively. The method proved to be reproducible, precise, and accurate as demonstrated by the intra-day and inter-day results obtained from QC urine samples of ZST316 and ZST152 (Table 4 and Table 5). Both ZST316 and ZST152 were stable at 37 °C in mice urine for at least 24 h and stored frozen at −20°C for one month. No chromatographic carry-over effect was observed injecting a blank sample after the highest standard calibration point.

### 2.3. Pharmacokinetics

**Figure 5:**
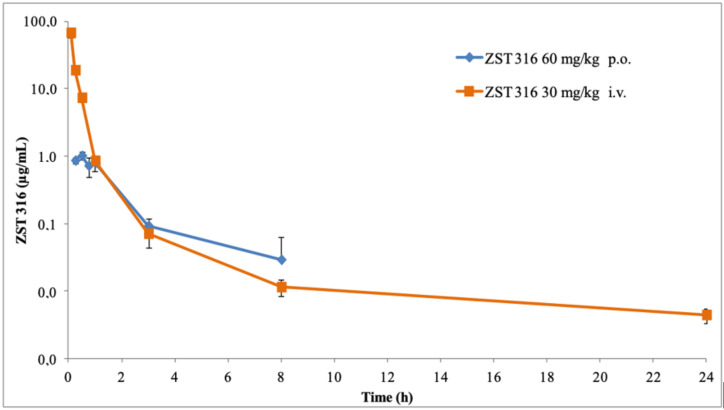
Plasma decay curves of ZST316 after single oral and intravenous administration.

**Figure 6:**
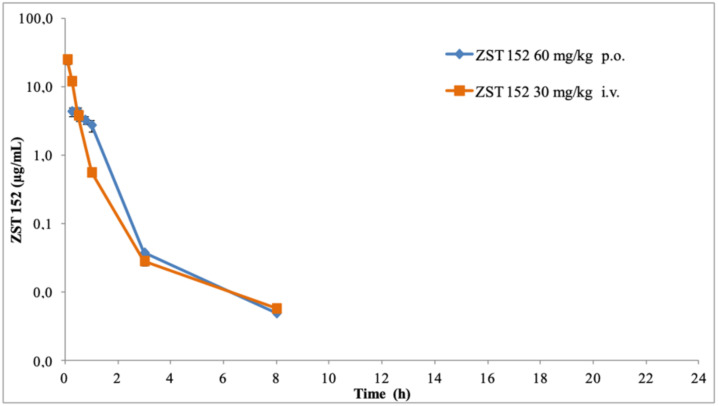
Plasma decay curves of ZST152 after single oral and intravenous administration.

The plasma pharmacokinetics of ZST316 and ZST152 after oral and intravenous administration and the derived parameters are shown in Figure 5 and Figure 6 and Table 6. After intravenous bolus administration of 30 mg/Kg, ZST316 achieved a Cmax of 67.4 µg/mL. Then, the compound was rapidly distributed and cleared from plasma with an elimination half-life of six hours. ZST316 was still detectable after 24 h at a concentration close to the LLOQ (i.e., 5 ng/mL) in two out of three samples. After oral administration, ZST316 achieved a Cmax of 1.02 µg/mL at 0.5 h and was measurable up to 8 h at a concentration of 0.029 µg/mL. ZST152, after intravenous administration of 30 mg/kg, achieved a Cmax of 24.9 µg/mL, distributed rapidly, and was cleared from plasma with an elimination half-life of 1.2 h. As a consequence of the relatively short half-life, ZST152 was detectable for up to 8 h at a concentration close to the LLOQ.

After oral gavage (60 mg/Kg), ZST152 peaked in plasma already at 15 min, achieving a Cmax of 1.65 µg/mL, which was higher than that of ZST316. ZST152, as also seen following the intravenous bolus, was measurable for up to 8 h at a concentration close to the LLOQ. The ratio of the calculated AUCs after oral and intravenous administration (normalized for the actual dose) indicated a bioavailability of 33% for ZST152 and less than 5% for ZST316. However, considering the actual plasma exposure after intravenous administration, the AUC of ZST316 was approximately twice that of ZST152 (Table 6).

The assessment of the urinary elimination after oral and intravenous administration, in the urine fraction collected 24 h post dose, is reported in Table 6. Regardless of the route of administration, the excretion of ZST152 was four- to fivefold that of ZST316 (12.5–22.2% vs. 2.3–7.5%). The relatively low amount of both compounds recovered in urine does not explain their accelerated clearance. To further investigate this issue, we searched for the presence of urinary metabolites in the 24 h urine samples in one mouse per treatment group. We identified at least eight molecules most likely originating from the parent compounds (Table 7; see Supplementary Figure S1 for the hypothesized structures). Their total amount suggests that metabolism plays an important role in the elimination of the compounds.

**Figure 7:**
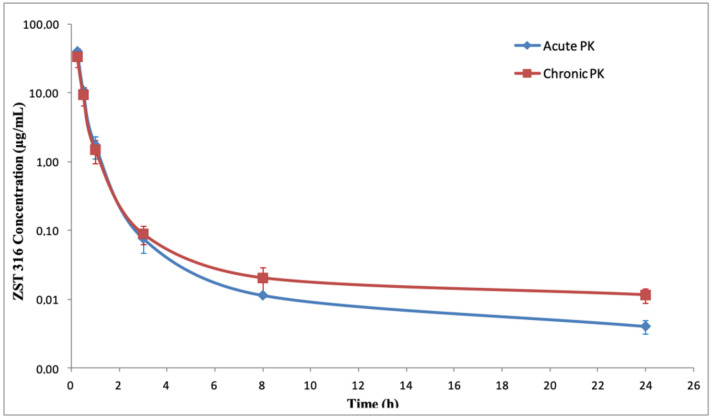
Plasma decay curves of ZST316 after acute and chronic intraperitoneal administration.

**Figure 8:**
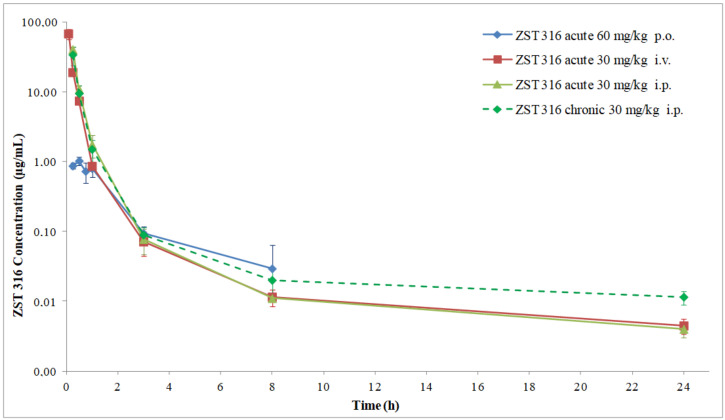
Comparison of ZST316 plasma decay curves after acute oral, intravenous, and intraperitoneal administration and chronic intraperitoneal administration.

The pharmacokinetics of the more potent DDAH1 inhibitor, ZST316, following acute and chronic intraperitoneal administration (single daily dose of 30 mg/Kg) and the derived parameters are shown in Figure 7 and Figure 8 (for a comparison with the intravenous and oral route) and Table 8. ZST316 peaked in plasma shortly after acute and chronic administration. The compound was eliminated from plasma with a half-life of 5.7 and 8.4 h after acute and chronic administration, respectively. No accumulation was observed, with acute and chronic AUC values of 14.45 and 12.57 µg/mL·h, respectively. The bioavailability of the intraperitoneal administration was 67% and 59% after acute and chronic treatment, respectively. The urinary excretion was 55% of the administered dose. Chronic treatment was well tolerated, and no clinical signs of toxicity or distress were noticed. As shown in Supplementary Figure S2, the body weight during treatment was similar to that of untreated animals.

## 3. Discussion

An accurate and reproducible bioanalytical HPLC-MS/MS method was developed and validated to measure the plasma and urine concentrations of the arginine analogues ZST316 and ZST152, inhibitors of human recombinant DDAH1, in mice. Using this method, a comprehensive pharmacokinetic assessment of the compounds was performed following acute and, for ZST316, chronic treatment to identify the appropriate route of administration and dosing regimen for in vivo efficacy studies. The higher oral bioavailability of ZST152 (33.3%) vs. ZST316 (4.7%) is likely due to the higher lipophilicity of the oxadiazolone moiety compared to the acylsulfonamide functional group, in line with what has been reported with other classes of drugs [24,25]. Furthermore, acylsulfonamides are commonly hydrolyzed to their carboxylic acid derivatives by hepatic carboxylesterase [26,27]. This would explain the high relative abundance of the metabolite M7 in urine after oral administration. Despite its relatively low oral bioavailability, the elimination half-life of compound ZST316 was markedly longer than that of ZST152. Furthermore, the chronic intraperitoneal administration of compound ZST316 was well tolerated, with a markedly higher bioavailability than after oral administration, at 59%, and no accumulation. Therefore, a once-daily intraperitoneal administration of ZST316 appears to be a suitable treatment regimen for in vivo efficacy studies targeting DDAH1. In particular, the recent demonstration by our group that DDAH1 inhibition with ZST316 suppresses in vitro vasculogenic mimicry and migration of MDA-MD-231 cells, an established triple-negative breast cancer cell line, warrants the investigation of these effects in xenograft studies. The intraperitoneal route is appropriate for this type of study as it is relatively simple, easy to manage, reproducible, suitable for chronic treatment, and, importantly, not associated with significant stress for the animal [28,29].

The relatively large volume of distribution following intravenous administration, 12.17 mL/Kg, suggests that compound ZST316 can effectively penetrate extra-vascular compartments. However, further studies are required to investigate the capacity of the compound to reach specific target sites, e.g., cancer tissues, that are characterized by neo-vascularization, erratic vascular networks, and development of resistance mechanisms reducing the exposure of cancer cells and the tumor microenvironment to chemotherapeutics and other drugs [30,31,32].

## 4. Materials and Methods

### 4.1. Compounds

ZST316 and ZST152 were provided by Flinders University (Adelaide, Australia). For analytical purposes, the compounds were dissolved in bi-distilled water and the solutions were used to develop the analytical method and for pharmacokinetic analysis. For treatment purposes, the compounds were dissolved in sterile water for injection (S.A.L.F. Bergamo, Italy).

### 4.2. Mice and Pharmacokinetic Study

Four-week-old female FVB mice were purchased from Charles River Italia (Calco, Italy). Animals were housed and handled under specific pathogen-free conditions in the Animal Care Facilities of the Istituto di Ricerche Farmacologiche Mario Negri. Under rigorous international standards, animals are regularly checked by a certified veterinarian who is responsible for health monitoring, animal welfare supervision, experimental protocols, and procedures revision. Procedures involving animals and their care were conducted in conformity with the following laws, regulations, and policies governing the care and use of laboratory animals: Italian Governing Law (D. lgs 26/2014; Authorization n.19/2008-A issued 6 March 2008 by the Ministry of Health); Mario Negri Institutional Regulations and Policies providing internal authorization for persons conducting animal experiments (Quality Management System Certificate—UNI EN ISO 9001:2008—Reg. N° 6121); the NIH Guide for the Care and Use of Laboratory Animals (2011 edition); EU directives and guidelines (EEC Council Directive 2010/63/UE); and the guidelines for the welfare and use of animals in cancer research [33].

Mice were treated with ZST316 and ZST152 using a dose of 60 mg/Kg (oral gavage) and 30 mg/Kg (intravenous bolus). For pharmacokinetic sampling, blood was collected at 15 and 30 min, and 1, 3, 8, and 24 h post dose. Three mice were analyzed per each time point. After the animals were anesthetized with isoflurane, the blood was drained from the retro-orbital plexus into heparinized tubes, and then centrifuged for 10 min at 4000× *g* at 4 °C to separate plasma. A 24-h urine fraction was collected in three mice for each drug and route of administration. Plasma and urine samples were stored at −20 °C until analysis by HPLC-MS/MS.

A subsequent study investigated the pharmacokinetics of acute and chronic intraperitoneal administration of the more potent DDAH1 inhibitor, ZST316 (30 mg/Kg). In this study, 18 mice received a single dose and were sampled within 24 h, and the other 18 were treated every day for three weeks (qdx21) and sampled on day 21. Mice were monitored daily for clinical signs of toxicity and distress, and body weight was measured at least twice a week. Blood samples were collected after both treatment regimens at 15 and 30 min, and 1, 3, 8, and 24 h post dose. Three mice were analyzed for each time point. After the animals were anesthetized with isoflurane, the blood was drained from the retro-orbital plexus into heparinized tubes, and then centrifuged for 10 min at 4000 g at 4 °C to separate plasma. A 24-h urine fraction was collected in three mice for each drug and route of administration. Plasma and urine samples were stored at −20 °C until analysis by HPLC-MS/MS.

### 4.3. Development of the Analytical Method

The analytical methods for ZST316 and ZST152 were similar as the compounds were mutually used as internal standards and fell into the same range of linearity. Therefore, the following sections describe the development of the method for compound ZST316.

#### 4.3.1. Preparation of Standard and Quality Control Plasma Samples

Control mice plasma (90 µL) was spiked with 10 μL of ZST316 working solutions in the range of 50–5000 ng/mL to obtain a final dilution of 1:10, giving six calibration standards into the range 5.0–500 ng/mL. The calibration curve included also a blank and a zero standard plasma sample (processed with IS). For quality control (QC) sample preparation, two fractions of mice plasma were mixed with an appropriate amount of QC working solutions to obtain QC plasma samples at the final concentration of 15 and 250 ng/mL.

#### 4.3.2. Extraction Procedure for Plasma Samples

In the preliminary phase of the method set up, we tried different solvents, 2-propanol, methanol (CH_3_OH), and acetonitrile (CH_3_CN) at different pH conditions, quickly obtaining a satisfactory recovery with CH_3_OH: 0.1% formic acid (HCOOH). Briefly, to assay ZST316, 10 µL of IS (ZST152 WS: 500 ng/mL) were added to 100 µL of plasma study samples, standards, or QCs in a polypropylene tube. The sample was then added with 10 µL of ammonium formate (NH_4_HCO_2_) 50 mM, mixed by vortex and added with 400 µL CH_3_OH: 0.1% HCOOH, vortex mixed again for 30 s and centrifuged at 13,200 rpm for 10 min at 4 °C. A volume of 450 µL of the supernatant was transferred to a new polypropylene tube, dried under nitrogen flow, and reconstituted with 100 µL of MP A:B (1:1, *v/v*). Finally, the mixed samples were centrifuged at 13,200 rpm for 10 min at 4 °C and the supernatant was transferred into glass vials for HPLC-MS/ MS analysis. Plasma samples containing drug concentrations above the highest standard point of the calibration curve were reanalyzed following dilution with the control matrix.

#### 4.3.3. Preparation of Standard and Quality Control Urine Samples

Control mice urine (45 µL) was spiked with 5 μL of working solutions in the range of 10–500 µg/mL to obtain a final dilution of 1:10, giving five calibration standards in the range 1–50 µg/mL. The calibration curve included also a blank and a zero standard urine sample (processed with IS). To prepare quality control (QC) samples, a fraction of mice urine was mixed with an appropriate amount of QC working solutions to obtain QC urine samples at the nominal concentration of 30 µg/mL.

#### 4.3.4. Extraction Procedure for Urine Samples

To assay ZST316, 5 µL of IS (ZST152 WS: 100 µg/mL) were added to 50 µL of urine samples, standards, or QCs in a polypropylene tube. After vortex mixing, 490 µL of MP A:B (1:1; *v/v*) were added to 10 µL of each sample to obtain a final dilution of 1:50. Samples were vortex mixed again for 30 s, centrifuged at 13,200 rpm for 10 min at 4 °C, and, finally, the supernatant was transferred into glass vials for HPLC-MS/ MS analysis. The urine samples with drug concentrations above the highest standard point of the calibration curve were reanalyzed following dilution with control matrix.

#### 4.3.5. HPLC-MS/MS Conditions

Reversed-phase chromatography was performed using a series 200 HPLC system (Perkin Elmer, Waltham, MA, USA) under gradient conditions with separation on a HILIC column, 3 µm, 100 A, 2.1 × 150 mm (Waters, Milford, MA, USA), coupled with a 3 µm, 3.9 × 5.0 mm guard column of the same material at the flow rate of 0.2 mL/min. Gradient features: 10% mobile phase (MP) A (NH_4_HCO_2_ 10 mM, 10% CH_3_CN, 0.1% HCOOH) and 90% MP B (CH_3_CN, 0.1% HCOOH) as the initial constant condition; then to 90% MP A in 6 min and the constant condition for 1.5 min. Return to the start condition in 0.5 min and conditioning for 5.5 min. Total run time: 13.5 min. Mass spectrometric detection was performed using an API 4000 triple quadrupole mass spectrometer (SCIEX, Framingham, MA, USA) supplied with a Turbo Ion Spray source set at 350 °C and −4200 V, analyzing biological samples with electrospray ionization (ESI) operating in negative ion mode. Purified air was used as nebulizer gas (Gas 1) and heater gas (Gas 2) and N_2_ was used as curtain gas and collision activated dissociation gas. They were set to 40, 50, 20, and 4 instrument units (psi), respectively. The declustering potential (DP) was set at −119 V and the collision exit potential (CXP) at –10 V. All parameters of the source were defined by direct infusion of ZST316 and ZST152 1 µg/mL in mobile phase B. The final quantification was carried out in Selected Reaction Monitoring (SRM) with the following transitions: m/z 307.9 → 208.0 (collision energy of −29.5 eV) for ZST316 and m/z 271.9 → 170.9 (collision energy −24.7 eV) for ZST152 (Figure 2 and Figure 3). Data were processed with the software package Analyst 1.6.2 (AB SCIEX).

### 4.4. Pharmacokinetic Analysis

Pharmacokinetic parameters and descriptive statistics (means, standard deviations, percentages) were calculated using Phoenix WinNonlin V 8.3 (Certara Inc., Princeton, NJ, USA).

## 5. Conclusions

The development and validation of a HPLC-MS/MS method for the determination of the DDAH1 inhibitors ZST316 and ZST152 in plasma and urine allowed for a comprehensive pharmacokinetic characterization of the compounds during acute and chronic treatment in mice. The results of this study permit the design of in vivo efficacy studies, using the appropriate route of administration and dosing regimens, to investigate the effects of pharmacological DDAH1 inhibition in specific experimental models of disease.

## Figures and Tables

**Table 1:** LLOQ of ZST316 and ZST152 in plasma.

	ZST316	ZST152
Actual LLOQ concentration (ng/mL)	5.0	5.0
Mean concentration found (ng/mL)	4.8 ± 0.3	5.3 ± 0.2
Accuracy %	95.8	106.1
Precision %	7.1	4.1

**Table 2:** Intra-day precision and accuracy results for ZST316 and ZST152 in plasma.

	ZST316		ZST152	
Actual Concentration (ng/mL)	15.0	250.0	15.0	250.0
Mean concentration found (ng/mL)	15.6 ± 0.91	237.6 ± 10.4	15.7 ± 0.78	228.6 ± 10.3
Accuracy %	103.7	95.0	104.9	91.4
Precision %	5.8	4.4	5.0	4.5
N	5	5	5	5

**Table 3:** Inter-day precision and accuracy results for ZST316 and ZST152 in plasma.

	Nominal Concentration (ng/mL)	
	15.0	250.0
ZST316	Measured Concentration	
Day 1	14.6	234.0
	16.4	256.0
	15.8	233.0
	16.4	231.0
	14.6	234.0
Day 2	21.6*	283.0
	15.5	271.0
	16.2	257.0
Day 3	19.6	279.0
	15.9	290.0
	16.8	284.0
Mean (*n* = 11)	16.2	259.3
SD	1.41	13.00
Precision (%)	8.7	5.0
Accuracy (%)	107.9	103.7
	**Nominal concentration (ng/mL)**	
	**15.0**	**250.0**
**ZST152**	**Measured concentration**	
Day 1	15.6	241.0
	15.8	229.0
	15.7	236.0
	14.7	216.0
	16.9	221.0
Day 2	15.9	258.0
	12.8	191.0 *
	15.6	236.0
Day 3	16.3	258.0
	18.2	256.0
	13.9	258.0
Mean (*n* = 11)	15.6	241.4
SD	1.44	16.9
Precision (%)	9.2	7.0
Accuracy (%)	103.9	96.6

* Value excluded from calculations because it was out of the acceptance criteria.

**Table 4:** Intra-day precision and accuracy results for ZST316 and ZST152 in urine.

	ZST316	ZST152
Actual concentration (µg/mL)	30.0	30.0
Mean concentration found (µg/mL)	33.3 ± 1.53	29.2 ± 1.1
Accuracy %	111.2	97.2
Precision %	4.6	3.8
N	3	3

**Table 5:** Inter-day precision and accuracy results for ZST316 and ZST152 in urine.

	Nominal Concentration (µg/mL)
	30.0
ZST316	Measured Concentration
Day 1	31.7
	33.7
	34.7
Day 2	34.2
	33.7
	27.7
Day 3	33.9
	44.9*
	29.7
Mean (*n* = 8)	32.4
SD	2.51
Precision (%)	7.7
Accuracy (%)	108.0
	**Nominal concentration** **(µg/mL)**
	**30.0**
**ZST152**	**Measured concentration**
Day 1	30.0
	29.6
	27.9
Day 2	28.8
	29.2
	N.D.*
Day 3	28.5
	30.5
	29.1
Mean (N=8)	29.2
SD	0.83
Precision (%)	2.8
Accuracy (%)	97.3

* Value excluded from calculations because it was out of the acceptance criteria.

**Table 6:** Pharmacokinetic parameters and urinary elimination of ZST316 and ZST152 after single oral and intravenous administration.

	ZST316 p.o. 60 mg/Kg	ZST316 i.v. 30 mg/Kg	ZST152 p.o. 60 mg/Kg	ZST152 i.v. 30 mg/Kg
Cmax (μg/mL)	1.02	67.4	4.34	24.9
Tmax (min)	30	5	15	5
AUC 0-8h (μg/mL·h)	1.94	21.4	6.17	9.25
AUC 0-last (μg/mL·h)	1.94	21.5	6.17	9.25
AUC 0-inf (μg/mL·h)	2.01	21.6	6.18	9.26
Half-life (h)	1.63	6.06	0.86	1.17
Clp (mL/h/Kg)	-	1.39	-	3.24
Vd (mL/Kg)	-	12.17	-	5.49
F %	4.7	-	33.3	-
24 h urinary excretion (% of dose)	2.3	7.5	12.5	22.2

Legend: p.o., oral gavage; i.v., intravenous; Cmax, peak concentration; Tmax, time to reach peak concentration; AUC, area under the curve; Cl_p_, plasma clearance; V_d_, volume of distribution; F, bioavailability.

**Table 7:** List of supposed metabolites (M) for ZST316 and ZST152 found in urine after oral and intravenous administration with quantification by HPLC MS/MS expressed as relative abundance to parent compound.

	ZST316				ZST152			
	Oral		Intravenous		Oral		Intravenous	
	Total Dose 1.308 mg Mouse #3		Tot. Dose 1.326 mg Mouse #1		Tot. Dose 1.158 mg Mouse #3		Tot. Dose 1.410 mg Mouse #1	
	Amount Recovered (µg)	% of Dose	Amount Recovered (µg)	% of Dose	Amount Recovered (µg)	% of Dose	Amount Recovered (µg)	% of Dose
ZST316	28.9	2.2%	41.2	3.1%	-	-	-	-
ZST152	-	-	-	-	75.0	6.5%	148	10.5%
M1	2.74	0.21%	0.23	0.017%	1.02	0.088%	0.55	0.039%
M2	-	-	-	-	0.27	0.023%	128	9.05%
M3	-	-	-	-	49.1	4.2%	2.62	0.19%
M4	108	8.2%	8.71	0.66%	87.8	7.6%	93.3	6.6%
M5	-	-	-	-	1.84	0.16%	298	21.2%
M6	-	-	-	-	0.16	0.014%	1.48	0.10%
M7	991	76%	86.1	6.5%	2.58	0.22%	1.77	0.13%
M8	53.4	4.1%	4.87	0.37%	-	-	-	-
M9	71.0	5.4%	4.28	0.32%	-	-	-	-

**Table 8:** Pharmacokinetic parameters and urinary elimination of ZST316 after acute and chronic intraperitoneal treatment.

	ZST316 Acute Treatment	ZST316 Chronic Treatment
Cmax (μg/mL)	40.2	33.6
Tmax (min)	0.25	0.25
AUC _0-8h_ (μg/mL·h)	14.34	12.32
AUC _0-last_ (μg/mL·h)	14.45	12.57
AUC _0-inf_ (μg/mL·h)	14.48	12.71
Half-life (h)	5.7	8.4
F %	67.2	58.9
24-h urinary excretion (% of dose)	56.1	54.1

Legend: Cmax, peak concentration; Tmax, time to reach peak concentration; AUC, area under the curve; F, bioavailability.

## Data Availability

The data supporting the reported results are available from the corresponding author upon request.

## Supplementary Materials

The following are available online. Figure S1: Hypothesized structures of the metabolites recovered in urine; Figure S2: Body weight in mice treated with intraperitoneal ZST316 (blue line) and untreated animals (red line).

## Sample Availability

Samples of the compounds are available from the authors.
