# Supplementary material for: Pharmacokinetic Characterization of the DDAH1 Inhibitors ZST316 and ZST152 in Mice Using a HPLC-MS/MS Method
Source: Molecules. 2022 Feb 2;27(3):1017. doi: 10.3390/molecules27031017 (PMC8840040; doi:10.3390/molecules27031017)
Supplement: Supplementary file 1 [file molecules-27-01017-s001.zip › molecules-1533427-supplementary.pdf]

Hypothesized structures of the metabolites recovered in urine

M1

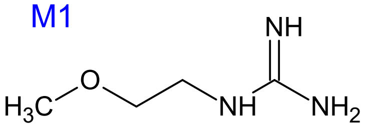

Molecular Formula: $C_4H_{11}N_3O$
Monoisotopic Mass: 117.090212 Da
[M+H]+: 118.097488 Da
[M-H]-: 116.082936 Da

M2

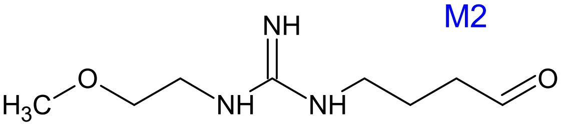

Molecular Formula: $C_8H_{17}N_3O_2$
Monoisotopic Mass: 187.132077 Da
[M+H]+: 188.139353 Da
[M-H]-: 186.1248 Da

M3

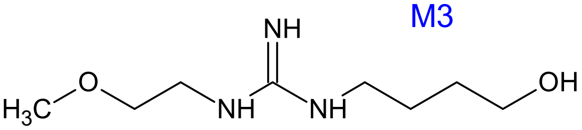

Molecular Formula: $C_8H_{19}N_3O_2$
Monoisotopic Mass: 189.147727 Da
[M+H]+: 190.155003 Da
[M-H]-: 188.14045 Da

M4

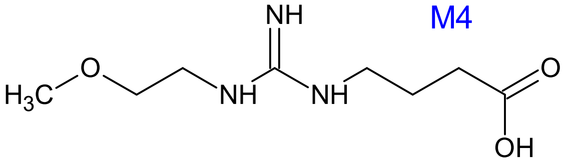

Molecular Formula: $C_8H_{17}N_3O_3$
Monoisotopic Mass: 203.126991 Da
[M+H]+: 204.134268 Da
[M-H]-: 202.119715 Da

M5

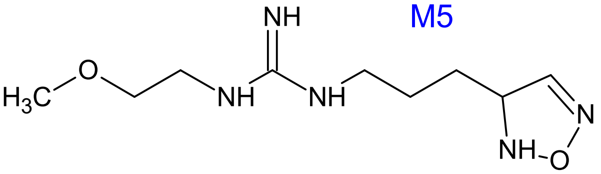

Molecular Formula: $C_9H_{19}N_5O_2$
Monoisotopic Mass: 229.153875 Da
[M+H]+: 230.161151 Da
[M-H]-: 228.146598 Da

M6

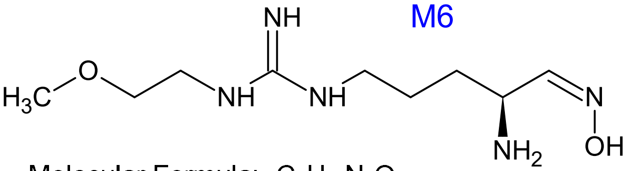

Molecular Formula: $C_9H_{21}N_5O_2$
Monoisotopic Mass: 231.169525 Da
[M+H]+: 232.176801 Da
[M-H]-: 230.162249 Da

M7

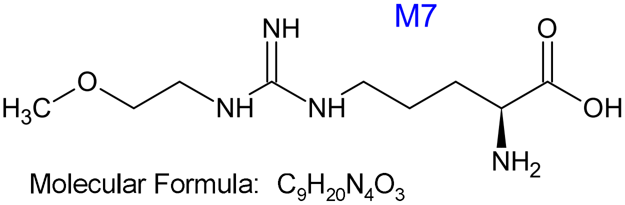

Molecular Formula: $C_9H_{20}N_4O_3$
Monoisotopic Mass: 232.153541 Da
[M+H]+: 233.160817 Da
[M-H]-: 231.146264 Da

M8

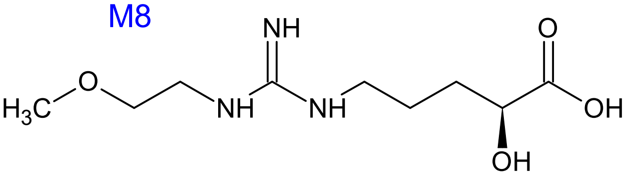

Molecular Formula: $C_9H_{19}N_3O_4$
Monoisotopic Mass: 233.137556 Da
[M+H]+: 234.144833 Da
[M-H]-: 232.13028 Da

M9

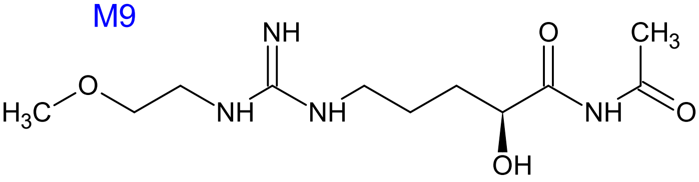

Molecular Formula: $C_{11}H_{22}N_4O_4$
Monoisotopic Mass: 274.164105 Da
[M+H]+: 275.171382 Da
[M-H]-: 273.156829 Da

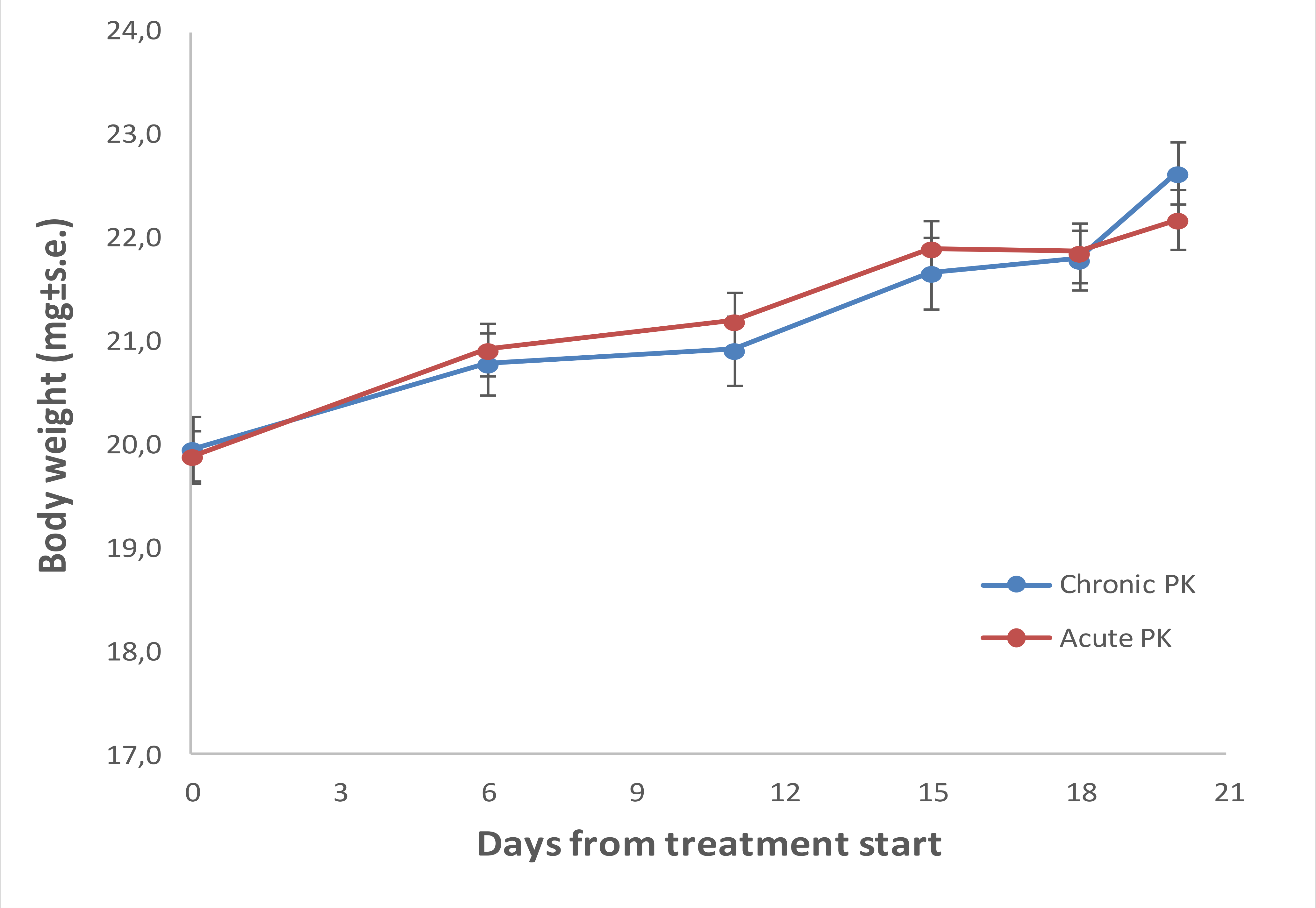

Body weight (mg±s.e.)
24,0
23,0
22,0
21,0
20,0
19,0
18,0
17,0
0
3
6
9
12
15
18
21
Days from treatment start
Chronic PK
Acute PK
